# Non-pharmacological treatments for chronic orchialgia: A systemic review

**DOI:** 10.1080/2090598X.2021.1958469

**Published:** 2021-08-04

**Authors:** Kareim Khalafalla, Mohamed Arafa, Haitham Elbardisi, Ahmad Majzoub

**Affiliations:** a Department of Urology, Hamad Medical Corporation, Doha, Qatar; b Department of Urology, Weill Cornell Medicine-Qatar, Doha, Qatar; c American Center for Reproductive Medicine, Cleveland Clinic, CL, OH, USA; d Andrology Department, Cario University, Cairo, Egypt

**Keywords:** Chronic orchialgia, microsurgical spermatic cord denervation, neuropathic pain, vasectomy reversal, orchidectomy

## Abstract

**Objective:**

: To review the outcomes of various therapeutic modalities that can be offered to patients with chronic orchialgia (CO) after failed conservative treatment.

**Methods:**

: A literature search was conducted using the PubMed and MEDLINE databases searching for articles exploring different CO treatment modalities. A Preferred Reporting Items for Systematic Reviews and Meta-Analyses approach was used to report the results of the literature search.

**Results:**

: A total of 34 studies were included for qualitative analysis. Most of the studies explored microsurgical spermatic cord denervation (MSCD; *n* = 19). Eight studies involved devices and interventions directed at blocking nerve sensations (pulsed radiofrequency stimulation, *n* = 5; transcutaneous electrical nerve stimulation, *n* = 1; cryoablation, *n* = 1; and mechanical vibratory stimulation, *n* = 1). Five studies reported on vasectomy reversal as a modality to relieve post-vasectomy pain syndrome (PVPS), while two studies explored the outcomes of orchidectomy on pain relief in patients with CO.

**Conclusion:**

: Several treatment methods are available in the urologist’s armamentarium for the treatment of CO. MSCD appears to be an appealing treatment modality with encouraging outcomes. Neuropathic pain can be managed with a number of relatively non-invasive modalities. Vasectomy reversal is a sound treatment approach for patients with PVPS and ultimately orchidectomy is a terminal approach that can be discussed with patients suffering from intractable pain.

## Introduction

Chronic orchialgia (CO) has been an escalating complaint in the past few years, commonly affecting young men and interfering with their daily and sexual activities. It is defined as continuous or intermittent, uni- or bilateral testicular discomfort of at least 3 months’ duration [1]. The challenges faced in managing patients with CO stem from the fact that various aetiologies may result in overlapping symptoms, and while several treatment modalities exist, they may have suboptimal outcomes.

The prevalence of CO has not been clearly defined; however, it is believed to have an increasing trend. In the United States, up to 100,000 men are diagnosed with CO each year [2]. While several causes, such as testicular infection or trauma, prior vasectomy, inguinal hernia surgery, recurrent epididymitis, varicoceles, hydroceles and prior scrotal/abdominal surgery have been associated with CO, in many of the cases (50%) the exact aetiology remains unknown [3–5].

The pathophysiology of CO is believed to be secondary to a phenomenon known as sensitisation of peripheral nerves that occurs after repeated noxious stimulation of nociceptors resulting in permanent changes in the peripheral as well as the central nervous system (CNS) [6]. The peripheral nervous system (PNS) and CNS are involved in the ‘wind-up’ phenomenon of chronic pain. The PNS neurones undergo modulation, which results in a decreased threshold for depolarisation, increased frequency of response and a decreased response latency time causing them to fire spontaneously after a while [7].

In the CNS, a similar process occurs, leading to phenotypic changes including up-regulation of intracellular cascade components and *N*-methyl-D-aspartate receptors. Even neurones adjacent to those involved in responding to the painful stimulus begin to fire on their own [7].

Treatment is mainly individualised and ranges from the more conservative medical measures such as NSAIDs, scrotal suspensions, antibiotics, antidepressants, anticonvulsants, physical therapy and acupuncture to the more invasive surgical interventions such as regional nerve block, epididymectomy, varicocelectomy, vasectomy reversal, hydrocelectomy and ending with epididymectomy and orchidectomy in intractable cases [8].

There is no doubt that CO is a medical condition that has a huge impact on patients’ lives and well-being. Most patients are persistent in seeking treatment and willing for invasive measures, especially when medical options are exhausted. Many surgical options exist, and none have been proven to be absolutely effective. The present review aimed at exploring the outcomes of various treatment modalities that have been proposed for patients with CO after exhausting all conservative attempts for pain relief.

## Methods

**Figure 1.**
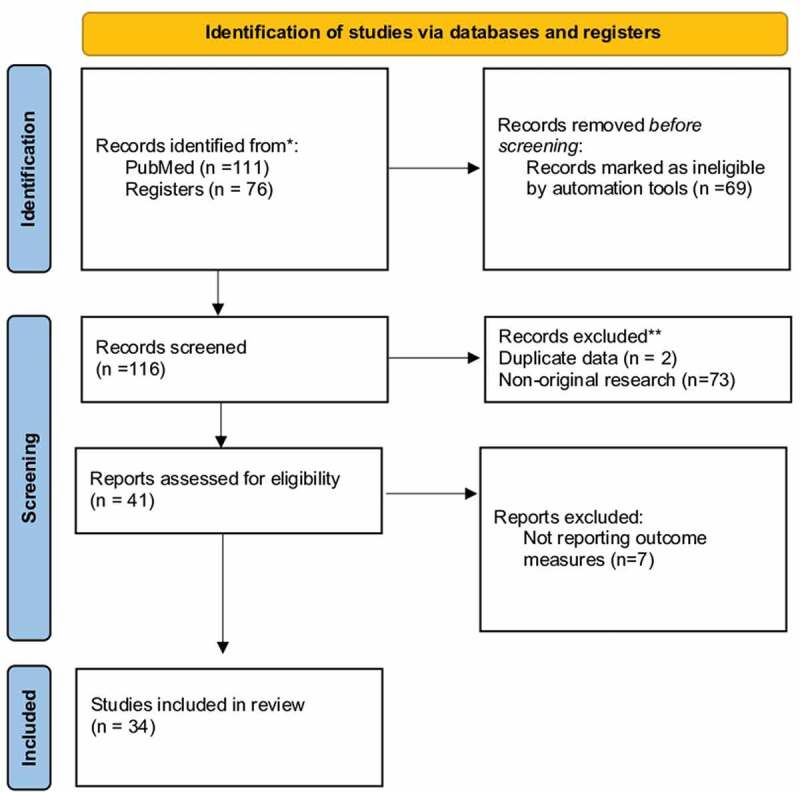
PRISMA flow chart

This review was conducted according to the Preferred Reporting Items for Systemic Reviews and Meta-Analyses (PRISMA) guidelines. The PubMed and MEDLINE databases were searched on 19 February 2021 using the keywords (‘chronic’) AND (‘orchalgia’ OR ‘orchialgia’) AND (‘treatment’ OR ‘management’ OR ‘surgery’ OR ‘injection’ OR ‘botox’ OR ‘cryoablation’ OR ‘block’ OR ‘denervation’)(Figure 1).

The inclusion criteria of the studies were patients with CO for ≥3-months duration together with failed conservative measures (including pharmacological treatment with/without scrotal support) for pain relief. The degree of pain improvement after the offered intervention was the primary outcome sought in this review.

Animal studies as well as studies written in a language other than English were excluded. Furthermore, reviews, case reports, commentaries and editorials were also excluded.

The obtained results were crosschecked by two authors (A.M. and K.K.). The titles and abstracts were then filtered in strict accordance with the inclusion and exclusion criteria. The full text of each article was subsequently reviewed to determine if the article would be included. Each article was checked by all authors to prevent inconsistency in findings. An Excel spreadsheet was created to organise and collect variables such as the study design, sample size, type of intervention performed, and the outcomes of intervention. Data were tabulated and frequencies of numerical values were presented as numbers (%).

## Results

The literature research retrieved a total of 187 articles. After applying the filters mentioned above, 116 studies were screened. In all, 82 studies were excluded due to duplicate data, being non-original research studies, and not meeting the primary objective of the review. Thus, 34 studies were included in this systemic review; six prospective, 21 retrospective and seven case series. Most studies reported the results of microsurgical spermatic cord denervation (MSCD) (*n* = 19) [9–27], while the remaining studies investigated the impact of various nerve-blocking techniques (*n* = 8) [28–35], vasectomy reversal (*n* = 5) [36–40], and extirpative surgery (*n* = 2) [41,42].

### Microsurgical spermatic cord denervation

Microsurgical spermatic cord denervation is the most commonly investigated treatment modality for alleviating the pain of idiopathic CO. The rationale behind the utilisation of this treatment option is based on blocking pain reception through obliterating nerve fibres traveling along the spermatic cord. Parekattil et al. [2] explored spermatic cord biopsy specimens to understand the distribution of the spermatic cord nerves looking for potential structural abnormalities along them. Specimens from 56 men with CO and 10 controls (varicocelectomy and orchidectomy) were examined and the authors revealed that an average of 25, 0.5 mm diameter, nerve fibres per patient were identifiable. Furthermore, a statistically significant prevalence of Wallerian degeneration was observed in men with CO in comparison to controls.

The present review identified 19 individual studies including 1676 testicular units for which MSCD was performed. In most cases an open approach for surgery was performed (inguinal [*n* = 14]; subinguinal [*n* = 3]; Table 1) [9–27]. Depending on the level of the incision, the aponeurosis of the external oblique muscle is either spared or opened. The ilioinguinal nerve is identified and a 2 cm segment is excised and ligated with proximal part buried well to avoid neuroma formation. Under microscopic magnification, the spermatic cord is brought up and its fascia is opened to expose the cord contents. Micro-Doppler ultrasonography (US) is used to identify the arterial flow in attempt to preserve testicular and cremasteric arteries during the procedure. The contents of the cord are ligated and dissected, which includes the cremasteric fascia, spermatic cord fat, and the pampiniform plexus of veins. Lymphatics are preferably spared to avoid hydrocele formation. The vas deferens is also preserved to reduce epididymal congestion, which decreases the incidence of post-vasectomy pain syndrome (PVPS). However, stripping of the perivasal tissues is performed to ensure obliteration of all the neural fibres.

**Table 1.** Studies exploring MSCD for the treatment of CO

Study	Study type	Sample size, *n* (testicular units)	Procedure approach	Method	Follow-up, months	Results	Complications
Marconi et al. 2015 [9]	Prospective	52	Inguinal	Microsurgery/ loops	6	80% pain free, 12% mild improvement, 8% no improvement	Haematocele (1.9%) Hydrocele (1.9%)
Larsen et al. 2013 [11]	Retrospective	70	Inguinal	Microsurgery	10	Overall pain reduction in 73%. Pain reduction in 67% of patients with previous surgical attempts (epididymectomy, varicocelectomy and orchidopexy). Pain reduction in 79% of patients with no previous surgery.	Low hanging testes (2.8%)
Oliveira et al. 2009 [12]	Retrospective	10	Inguinal	Microsurgery	24	70% pain free, 20% mild improvement, 10% no improvement	–
Cassidy et al. 2015 [16]	Case series	9	Inguinal	Microsurgery	9	77% pain free, 22% mild improvement	–
Strom et al. 2008 [14]	Retrospective	95	Inguinal	Microsurgery	20	71% pain free, 17% mild improvement, 12% no improvement	Hydrocele (2.1%) Testis atrophy (2.1%) Wound infection, seroma haematoma (4.2%)
Levine et al. 1996 [17]	Case series	8	Inguinal	Microsurgery	36	87.5% pain free, 12.5% mild improvement	Hydrocele (12.5%) Testis atrophy (12.5%) Seroma (12.5%)
Levine et al. 2001 [13]	Retrospective	33	Inguinal	Microsurgery	20	76% pain free, 9.1% mild improvement, 15% no improvement	Hydrocele (3%) Testis atrophy (6%) Wound infection, seroma (6%)
Calixte et al. 2017 [18]	Retrospective	860	Subinguinal	Robotic	48	49% pain free, 34% mild improvement, 17% no improvement	Hydrocele (0.1%) Wound infection, seroma, haematoma, dehiscence (5%)
Chaudhari et al. 2019 [15]	Prospective	62	Inguinal	Microsurgery	24	81.5% pain free, 10.5% mild improvement, 7.9% no improvement	Hydrocele (3.2%) Wound infection, seroma, haematoma, dehiscence (9.6%)
Goedde et al. 2020 [19]	Case series	3	Subinguinal	Robotic	5	100% pain free	–
Long et al. 2019 [20]	Retrospective	28	Subinguinal	Microsurgery	24	53.5% pain free, 36.7% mild improvement, 9.8% no improvement	–
Murthy et al. 2020 [21]	Retrospective	103	Subinguinal	Microsurgery	3	73% success rate.	Hydrocele (1.9%) Wound infection (3.8%)
Ahmed et al. 1997 [22]	Prospective	17	Inguinal	–	–	76% pain free, 24% mild improvement	–
Choa and Swami 1992 [23]	Case series	4	Inguinal	-	18.5	100% pain free	–
Heidenreich et al 2002 [24]	Retrospective	35	Inguinal	Microsurgery/ loops	34	96% pain free, 4% mild improvement	Haematoma (5.7%)
Kavoussi et al. 2020 [25]	Retrospective	143	Inguinal	Microsurgery	12	67.8% pain free, 36.7% mild improvement, 9.8% no improvement	–
Benson et al. 2013 [10]	Retrospective	77	–	–	10	73% improvement rate	–
Cadeddu et al. 2012 [26]	Retrospective	9	Retroperitoneal	Laparoscopic	25.1	71% improvement rate	Hydrocele (11.1%)
Oomen et al. 2014 [27]	Retrospective	58	Inguinal	Microsurgery	42.8	52% pain free, 34% mild improvement, 14% no improvement	Hydrocele (1.7%) Testicular atrophy (1.7%)

Most of the included studies utilised a visual analogue scale (VAS) for pain to monitor the outcome of surgery. With a follow-up duration of up to 48 months; overall, the reported pain-free status after surgery was between 52% and 100%. Side effects were minimal and included hydrocele (0.1–12.5%), haematocele (1.9%), testis atrophy (2.1–12.5%), and other wound-related complications.

Almost all the included studies performed a spermatic cord block treatment with a long acting or long- + short-acting local anaesthetic agent prior to the definitive MSCD procedure. The available evidence indicates that the patient response to the spermatic cord bock is an independent predictor of MSCD success rate. A retrospective study by Benson et al. [10] particularly explored this association revealing a significant impact for the improvement in the VAS pain score following spermatic cord block on the MSCD outcome (coefficient 0.4 ± 0.19, *P* = 0.03). Out of the 71 patients investigated, a durable response to MSCD was observed in 13 (57.3%) patients who had a 50–75% improvement after the cord block and in 57 (74.6%) there was a 76–100% improvement after the cord block. In another study by Kavoussi et al. [25], the aetiology of orchialgia appeared to be a significant predictor of MSCD failure. In their cohort, in comparison to patients with PVPS, the authors observed a lower odds for failure by 79% in patients with idiopathic CO and by 83% in patients with prior scrotal/inguinal surgery.

### *Devices inhibiting nerve sensations (* Table 2 *) [28–42]*

Several treatment modalities directed at inhibiting or obliterating the sensory impulses from the testis have been investigated. These include nerve blocks with anaesthetics and steroids, cryoablation, radiofrequency and transcutaneous vibratory or electrical nerve stimulation.

**Table 2.** Studies exploring interventions to inhibit nerve sensations, vasectomy reversal and extirpative surgery for the management of CO

Study	Design	Sample size, *n*	Procedure	Measuring scale	Outcome
Tantawy et al. 2017 [28]	Prospective comparative study	71; Group A (*n*= 36) received TENS+ analgesia, Group B (*n*= 35) received analgesia	Self-adhesive electrodes, the anode electrode was placed on the lower abdomen (suprapubic area medial to the iliofemoral ligament) in the area with the highest pain and the cathode electrode placed 5 cm proximal to the anode in relation to the trunk side.	VAS and QoL	A significant reduction in pain and QoL after intervention and at 2-month follow-up in Group A (*P* < 0.001 for all)
Hetta et al. 2018 [29]	Prospective randomised, controlled clinical trial.	70; PRF group (*n* = 35), Sham group (*n* = 35)	PRF to the ilioinguinal nerve and genital branch of the genitofemoral nerve	VAS and GPE	The percentage of patients that showed >50% reduction of their VAS pain score was 80% (24/30) in the PRF group vs 23.33% (7/30) in the sham group. There was a significant reduction of the mean post-procedural VAS pain score at 2, 4, 6, 8, and 12 weeks (*P* = 0.001) in the PRF group in comparison to the sham group.
Cohen and Foster 2003 [30]	Case series	3	PRF to the ilioinguinal nerve and genital branch of the genitofemoral nerve	Self-report	All patients reported complete pain relief after 6 months
Rozen and Parvez 2006 [31]	Case series	5	PRF to T12, L1 and L2 nerve root	Self-report	All patients reported 75–100% pain relief after 6–9 months
Venkataparthasarathy and Ather, 2006 [32]	Retrospective	15	PRF to ilioinguinal/ genitofemoral nerves	VAS	Average VAS score was 7.5 pre-procedure, 4.3 immediate post-procedure, 2.8 at 6 weeks and 3.2 at 6 months.
Basal et al. 2012 [33]	Case series	6	PRF applied into the spermatic cord 2 cm distal to the external ring	VAS	Mean VAS pain score before and after the procedure were 9 and 1, respectively. None of the patients needed any analgesics after the procedure or during the follow-up period. Mean (SD) follow-up period was 20 (2.5) weeks. No recurrence was noted, and none of the patients needed additional therapy.
Khandwala et al. 2017 [34]	Prospective	10	Topical ball vibratory stimulation to the external ring 20 min/day for 4 weeks	VAS	Average pain score decreased from 4.9 to 2.7 (*P* = 0.009), while maximum pain severity decreased from 6.3 to 4.0 (*P* = 0.013). The frequency of pain also decreased for 55.6% (5/9) of men.
Calixte et al. 2019 [35]	Retrospective	221	Cryoablation medial and lateral to the spermatic cord at the level of the external ring	VAS and PIQ-6	75% significant reduction in pain (11% complete resolution and 64% ≥50% reduction in pain). Objective PIQ-6 outcomes: 53% significant reduction at 1 month (279 cases), 55% at 3 months (279 cases), 60% at 6 months (279 cases), 63% at 1 year (279 cases), 65% at 2 years (275 cases), 64% at 3 years (232 cases), 59% at 4 years (128 cases), and 64% at 5 years (53 cases) postoperatively.
Myers et al. 1997 [36]	Retrospective	32	Vasovasostomy	Self-report	Overall, 27 of 32 men had resolution of pain.
Nangia et al. 2000 [37]	Retrospective	13	Vasovasostomy	Self-report	Postoperatively 9 of the 13 men (69%) became completely pain-free. Mean follow-up was 1.5 years.
Lee et al. 2012 [38]	Retrospective	32	Vasovasostomy	VAS and specially designed questionnaire	The mean (SD) VAS pain score was 6.64 (1.00) preoperatively and 1.14 (0.71) postoperatively (*P* < 0.001). The difference in the mean (SD, range) pre- and postoperative VAS pain score was 6.00 (1.25, 4–8) in the patency group and 4.43 (0.98, 3–6) in the no patency group (*P* = 0.011). A significant difference in procedural satisfaction with surgical outcome was observed between patency and no patency groups (*P* = 0.014).
Horovitz et al. 2012 [39]	Retrospective	23	Vasovasostomy	Self-report and QoL	Improvement of pain occurred in 93% (13 of 14) and 50% were rendered pain-free, with an average improvement in pain intensity scores of 65% (*P* < 0.005). 15% (2 of 13) had a recurrence of pain to baseline but overall, 79% (11 of 14) had a durable positive response. QoL was significantly improved after vasovasostomy (*P* < 0.005) and 93% (13 of 14) of the patients said they would undergo the same operation again.
Polackwich et al. 2015 [40]	Retrospective	31	Vasovasostomy	Specially designed survey	82% of patients reported improvement in their pain at a mean (SD) 3.2 (3.4) months after vasectomy reversal. 34% patients had complete resolution of all pain. Mean (SD) pain score before procedure was 6.4 (2.4), decreasing to 2.7 (2.7) afterward. There was a 59% improvement in pain scores (*P* < 0.001).
Davis et al. 1990 [41]	Retrospective	34	Orchidectomy (24) Epididymectomy (10) Orchidopexy (5) Hydrocelectomy (1)	Self-report	Orchidectomy: complete relief (16, 66.7%); partial relief (7, 29.2%); no relief (1, 4.2%) Epididymectomy: 9/10 further underwent orchidectomy
Yamamoto et al. 1995 [42]	Retrospective	12	Orchidectomy (4) Spermatic cord block (3) Bilateral transrectal injections of local anaesthetic (2)	Self-report	Orchidectomy: complete relief (3, 75%); partial relief (1, 25%)

GPE: global perceived effect.QoL: Quality of life; VAS: visual analogue scale;PRF: pulsed radiofrequency;TENS: transcutaneous electrical nerve stimulation;PIQ-6: pain impact questionnair

### Pulsed radiofrequency stimulation (PRF)

Pulsed radiofrequency stimulation is a well-established treatment modality for a number of neurogenic and non-neurogenic painful conditions. It is a non-nerve destructive, minimally invasive modality in which electrical pulses are released from the tip of an electrode to create an impulse without causing thermal injury [43]. Unlike ablation procedures, PRF interrupts pain signals through biological changes rather than nerve destruction ultimately reducing pain sensation from the affected area [43]. PRF was investigated in five studies including 99 patients. In the majority of cases the impulses were applied to the ilioinguinal nerve or the genital branch of the genitofemoral nerve with the exception of the study by Rozen and Parvez [31] who performed the procedure on T12, L1–2 nerve roots. The probe is inserted into the desired area through fluoroscopic or US guidance and stimulation is reportedly performed with the following settings: 2–60 V, 40–50 Hz with impulses every 20 ms. The most informative study was that of Hetta et al. [29], who performed a double-blind, sham-controlled, clinical trial in which 70 patients were randomized to receive PRF or sham intervention. The patients were followed with a VAS for pain and 80% of those receiving PRF had >50% reduction in VAS pain score vs 23.3% in the sham group. The other reports were mostly case series [30,31,33], except for a single retrospective study [32], and all reported pain reduction in most of their patients that was maintained for a follow-up of 6–9 months. PRF could be an appealing intervention, especially that it is less invasive than surgery, and can be particularly helpful for neuropathic pain secondary to nerve entrapment originating from previous inguinal surgery.

### Transcutaneous electrical nerve stimulation (TENS)

Transcutaneous electrical nerve stimulation is an electro-analgesia modality that is non-pharmacological and non-invasive, and which has been utilised in several chronic painful conditions [44,45]. It is hypothesised that TENS illicit pain relief through central and peripheral mechanisms [46]. The procedure entails delivery of electrical impulses across the skin that activate underlying nerve structures. This may interfere with peripheral impulses overwhelming afferent sensations ‘busy line effect’, thereby blocking pain [46]. Centrally, it can reduce nociceptor cell activity of the spinal cord and activate brain descending inhibitory pathways [46]. The effects of TENS are mediated through a number of receptors in the CNS and PNS including opioid, serotonin, muscarinic and α_2_-adrenergic receptors [46].

A single randomised clinical trial by Tantawy et al. [28] was conducted on 71 patients with CO who were randomised into a study group (analgesia + TENS, *n* = 36) and a control group (analgesia alone, *n* = 35). The TENS was performed five-times per week for 4 weeks, where the anode electrode was placed in the suprapubic area medial to the iliofemoral ligament and the cathode 5 cm proximal to it. A mean TENS intensity of 25 mA was used. The results revealed that a significant reduction in the VAS pain score was noted only in the study group (mean [SD] 7.35 [1.13] vs 4.45 [0.88], *P < *0.001) 2 months after the intervention and not in the control group. Furthermore, a significant improvement in patients’ quality of life (QoL) was also only reported by the study group.

### Cryoablation

Cryoablation is a treatment modality that utilises low temperatures to obliterate nerve fibres thereby blocking pain sensation. It has been utilised for a long time particularly for nerve entrapments [47]. In the setting of CO, cryoablation has been investigated in a single study by Calixte et al. [35] particularly for patients with persistent pain following MSCD. The authors speculate that failure of pain relief in this subset of patients may be due the presence of residual nerves medial and lateral to the spermatic cord at the level of the external ring. As such, the authors performed US-guided cryoablation o 221 patients by inserting the cryoprobe medial and lateral to the spermatic cord at the external ring. Argon gas was used to achieve a temperature of – 106.7°C (–160 °F) at the probe tip. Two freezing cycles of 90 s each were performed with passive thawing until a 1.5-cm ice ball was visualised through real-time US on either side of the spermatic cord. Patients were followed with a VAS pain score and the Pain Impact Questionnaire-6 (PIQ-6). The authors reported 75% pain reduction with the VAS following the procedure (11% complete resolution and 64% ≥50% reduction in pain). The results of the PIQ-6 questionnaire further confirmed that a favourable response can be achieved for a prolonged period, as 64% had a significant reduction of pain 5 years after the intervention (*P* < 0.001). The side effects were minimal and included wound infection in two patients and penile pain in four.

### Mechanical vibratory stimulation

Vibratory treatment is another non-invasive therapeutic modality implemented in non-urological painful conditions such as fibromyalgia, muscle pain and diabetic neuropathy [48,49]. Mechanical vibratory stimulation is believed to activate mechanoreceptors and competitively inhibit nociceptors in the PNS and CNS [50]. This treatment modality was utilised in a single study for the treatment of CO [34]. The authors utilised a battery-operated massage ball to apply vibratory stimulation on the external rings of the ipsilateral testes of nine patients. Patients were instructed to use the devise for 20 min per day for 4 weeks. A reduction in the average (–2.2, *P* = 0.009) and maximum (–2.3, *P* = 0.013) daily pain was observed in 78% of patients. A reduction in the frequency of pain was also reported by 56% of patients.

Mechanical and electrical stimulation techniques are promising non-invasive treatment methods with no risk profile. However, further studies are still required to demonstrate their benefit in clinical practice.

### Vasectomy reversal

A distinct subset of patients with CO are those who develop their symptoms following a vasectomy procedure for elective sterilisation. Chronic pain following this procedure, termed PVPS, has been identified as a late complication occurring in up to 15% of cases [51]. It is defined as intermittent or constant scrotal pain that occurs after a vasectomy procedure and stays for >3 months. The pain is typically aggravated with ejaculation, physical activity, and with pressure over the testis. Conservative measures of treatment can be tried first; however, if the pain persists for a long duration and affects the patients’ daily activities, then a vasectomy reversal procedure should be considered. A total of five studies including 131 patients who underwent vasectomy reversal due to PVPS were identified [36–40]. Overall, the reported improvement in pain after surgery was 69–93%. Lee et al. [38] linked pain improvement with the patency rates after surgery. In all, 22 patients who underwent vasectomy reversal for PVPS completed a study questionnaire and were assessed with a VAS pain score before and after the operation. The patency rate was 68.2% and the pain reduction was significantly more meaningful in the patent group, with a VAS mean (SD) difference of 6.0 (1.25) vs 4.43 (0.98) in the non-patent group (*P* = 0.014). This result highlights the relationship between vasectomy and the development of pain after the procedure and hints that an obstructive pathophysiology is the most likely mechanism for PVPS.

### Extirpative surgery

A proportion of patients with CO may not benefit from any form of treatment. An integrative literature review by Quallich et al. [52] revealed that ~18.6% of patients would never receive a satisfactory explanation for their pain and would remain symptomatic even after consulting with an average of 4.5 urologists and after undergoing 4.7–7.2 procedures. Therefore, in a small number of patients, orchidectomy may be discussed as a final resolution to overcome their symptoms. However, such cases would require appropriate counselling for the possible risks and complications of surgery including hypogonadism, sexual implications, and psychological alterations that may develop due to the cosmetic appearance of the genitalia. Furthermore, patients should be informed that pain relief may not by achieved 100% of the time. In fact persistent neuropathic pain, also known as ‘phantom pain’ has been reported in up to 25% of patient after undergoing orchidectomy for testicular cancer [53]. The present review identified two studies in which the outcome of orchidectomy in patients with CO was reported. Davis et al. [41] performed a retrospective study of 34 patients, 24 of whom had undergone orchidectomy. After surgery, complete pain relief was achieved in 16 patients (66.7%), while seven patients had partial relief of pain (29.7%) and one patient had persistent pain. Of the 24 orchidectomies performed 15 were performed through an inguinal approach, while the remaining nine surgeries were through a scrotal approach. The authors reported better pain relief in patients who underwent inguinal (73%) vs scrotal (55%) orchidectomy. Similarly, Yamamoto et al. [42] reported the outcome of orchidectomy in four patients, revealing that complete pain relief was noted in three and partial relief in one. These results indicate that complete pain relief cannot be guaranteed in all cases and proper patient counselling, preferably with preoperative psychological assessment, would be beneficial before undergoing such a procedure.

## Conclusions

Chronic orchialgia is a debilitating clinical condition with a significant impact on patients’ wellbeing. A variety of therapeutic modalities can be offered to patients with persistent pain despite conservative treatment attempts. MDSC is an effective treatment option with success rates ranging between 52% and 100%. A more favourable outcome can be expected in patients having a positive response to preoperative spermatic cord block and in patients with idiopathic CO. Patients who fail MSCD may be offered several nerve-blocking devices or interventions including PRF, cryoablation, TENS, and mechanical vibratory stimulation. Persistent pain despite all measures is not uncommon and, in such cases, orchidectomy may be a final resort. However, adequate counselling and psychological support is required before undergoing such an invasive approach.
